# The past, present and future of penile cancer in the Nordic countries. Results from NORDCAN

**DOI:** 10.1002/bco2.70041

**Published:** 2025-06-27

**Authors:** Christian Arvei Moen, Axel Gerdtsson, Mikael Aagaard, Andreas Hopland, Peter Kirrander, Jakob Kristian Jakobsen

**Affiliations:** ^1^ Department of Urology Haukeland University Hospital Bergen Norway; ^2^ Department of Clinical Medicine University of Bergen Bergen Norway; ^3^ Department of Urology Skåne University Hospital Malmö Sweden; ^4^ Department of Translational Medicine Lund University Lund Sweden; ^5^ Department of Urology Rigshospitalet University Hospital Copenhagen Denmark; ^6^ Department of Urology Oslo University Hospital Oslo Norway; ^7^ Department of Urology, Faculty of Medicine and Health Örebro University Örebro Sweden; ^8^ Department of Urology Aarhus University Hospital Aarhus Denmark; ^9^ Department of Clinical Medicine Aarhus University Aarhus Denmark

**Keywords:** incidence, NORDCAN database, penile cancer, survival, time trends

## Abstract

**Objectives:**

This work aims to present epidemiological data on penile cancer from the Nordic region (Denmark, Finland, Iceland, Norway, Sweden) and discuss the results in light of the present knowledge and possible future treatment strategies.

**Patients and Methods:**

All patients diagnosed with penile cancer in the Nordic countries between 2000 and 2022 were identified in the NORDCAN database. Data on the number of new cases per year, incidence (presented as an age‐standardized rate using the Nordic population as a reference), prevalence, mortality, relative survival and predictions on the future societal impact of this cancer was retrieved.

**Results:**

A total of 6106 cases of penile cancer were registered in a population that increased from about 12–14 million men over the period. The age‐standardized incidence has significantly increased over time and is now above 2.0 per 100 000 men. Currently, more than 300 new cases are diagnosed each year, and more than 3000 men live with a penile cancer diagnosis. Mortality, as well as both 1‐year and 5‐year relative survival have, however, remained nearly unchanged. Using the NORPRED model, it is predicted that the yearly number of penile cancer cases will continue to increase in all the countries investigated over the next decade.

**Conclusion:**

Penile cancer incidence and prevalence have increased in the Nordic region over the last 20 years. Mortality and survival, however, have remained unchanged. The number of new penile cancer cases is predicted to increase over the next decade. Better treatment options for these patients are therefore urgently needed.

## 1 INTRODUCTION

Penile cancer is a rare disease with a referenced incidence below 1.0 per 100 000 men in Europe.^1^ Surgical removal of the penile tumour and, if necessary, regional lymph node metastases remains the hallmark of the primary treatment strategy. In the metastatic setting, first‐line oncological treatment with radiotherapy and/or chemotherapy regimens or second‐line therapy using immune‐checkpoint inhibitors may be appropriate,^2^, ^3^ but the prognosis in advanced penile cancer remains poor.^1^

The Nordic countries include Denmark, Finland, Iceland, Norway and Sweden. The Nordic region also includes the Faroe Islands and Greenland. The cancer registries from these countries/regions monitor the incidence, prevalence, mortality and survival of cancers over time, and these parameters can also be used to make predictions on the future societal impact of specific cancers. Moreover, all these registries collaborate and deliver data on cancer statistics to the NORDCAN database.^4^ The database has its own homepage (https://nordcan.iarc.fr/en), where results of common epidemiological variables can be freely accessed and studied in an interactive manner.^5^ The Nordic countries all have a similar public national health care system, providing universal coverage for the entire population. Furthermore, for penile cancer specifically, care was centralized to one or a few centres in each country over the period 2010–2016. All patients are therefore currently treated at dedicated centres by personnel skilled in the treatment of penile cancer. Surgeons from these centres participate in a collaborative network, the Scandinavian penile cancer group (SCAPECA), which functions under the auspices of the Scandinavian Association of Urology. Thus, comparisons across the different countries are reasonable, intriguing and relatively straight forward.

The aim of this study is to present epidemiological data on penile cancer from the NORDCAN database and discuss the results considering the present knowledge and possible future treatment strategies.

## 2 PATIENTS AND METHODS

### 2.1 Data acquisition

The NORDCAN database homepage was accessed in February of 2025 (Data version: 9.4 – 07.24).^6^ Data on the number of yearly cases of penile cancer and the predicted number of yearly future cases, incidence, mortality and prevalence as well as relative 1‐ and 5‐year survival estimates were investigated for all the NORDCAN countries combined as well as for each separate country. For each variable of interest, the data were downloaded as an excel file and read into R version 4.3.3.^7^

### 2.2 Illustrations

All figures were recreated using the package ‘ggplot2’.^8^ For variables available on a yearly basis, data are presented over the period 2000–2022. For variables available over a 5‐year period, the 20‐year period 2003–2022 was chosen. Curves were smoothed when applicable for easy visualization of the underlying trend. Smoothing was performed by using the loess function (with span = 1 and degree = 1) from the built‐in ‘stats’ package in R. The only exception to this rule was analysis of data on future predictions of the number of penile cancer cases in each country. For this variable, the span parameter was set to 0.2, to better visualize the accurate predictions. The resulting smoothed values were then plotted by ‘ggplot2’.

### 2.3 Details regarding the NORDCAN variables

To describe the magnitude of the change in the trend of the age‐standardized rates (ASR) for incidence in NORDCAN, the estimated annual percentage change (EAPC) was used.^9^

For survival estimates, the relative survival in NORDCAN was estimated using the Pohar Perme estimator, which results in a net survival estimate.^10^

Predictions regarding the number of cases that will be diagnosed in each country over the next 20 years are based on default settings of the NORDPRED model.^11^

Due to the low number of cases diagnosed in Iceland (only 66 cases between 2000 and 2022), these numbers were included in the presented NORDCAN data but not included when plots for individual countries are presented. Furthermore, patients from the Faroe Islands and Greenland treated with curative intent are most often treated in Denmark and will be included in the Danish data.

## 3 RESULTS

The population of men in the Nordic countries increased from around 12 million in 2000 to around 14 million in 2022. A total of 6106 cases of penile cancer were registered over the period. A detailed breakdown of numbers for each country/region is presented in Table 1.

**TABLE 1:** Population of men and number of penile cancer cases for each country/region of the Nordic countries over the period 2000–2022.

Countries/region	Population (2000)	Population (2022)	Cases (2000–2022)
Nordcan countries^a^	11 965 320	13 971 467	6106
Sweden	4 386 439	5 279 520	2509
Denmark	2 634 122	2 937 921	1446
Norway	2 224 223	2 751 407	1207
Finland	2 526 188	2 748 293	852
Iceland	140 713	196 504	66

^a^ In addition to the specific countries above, the Nordcan countries also include data from the Faroe Islands and Greenland.

**FIGURE 1:**
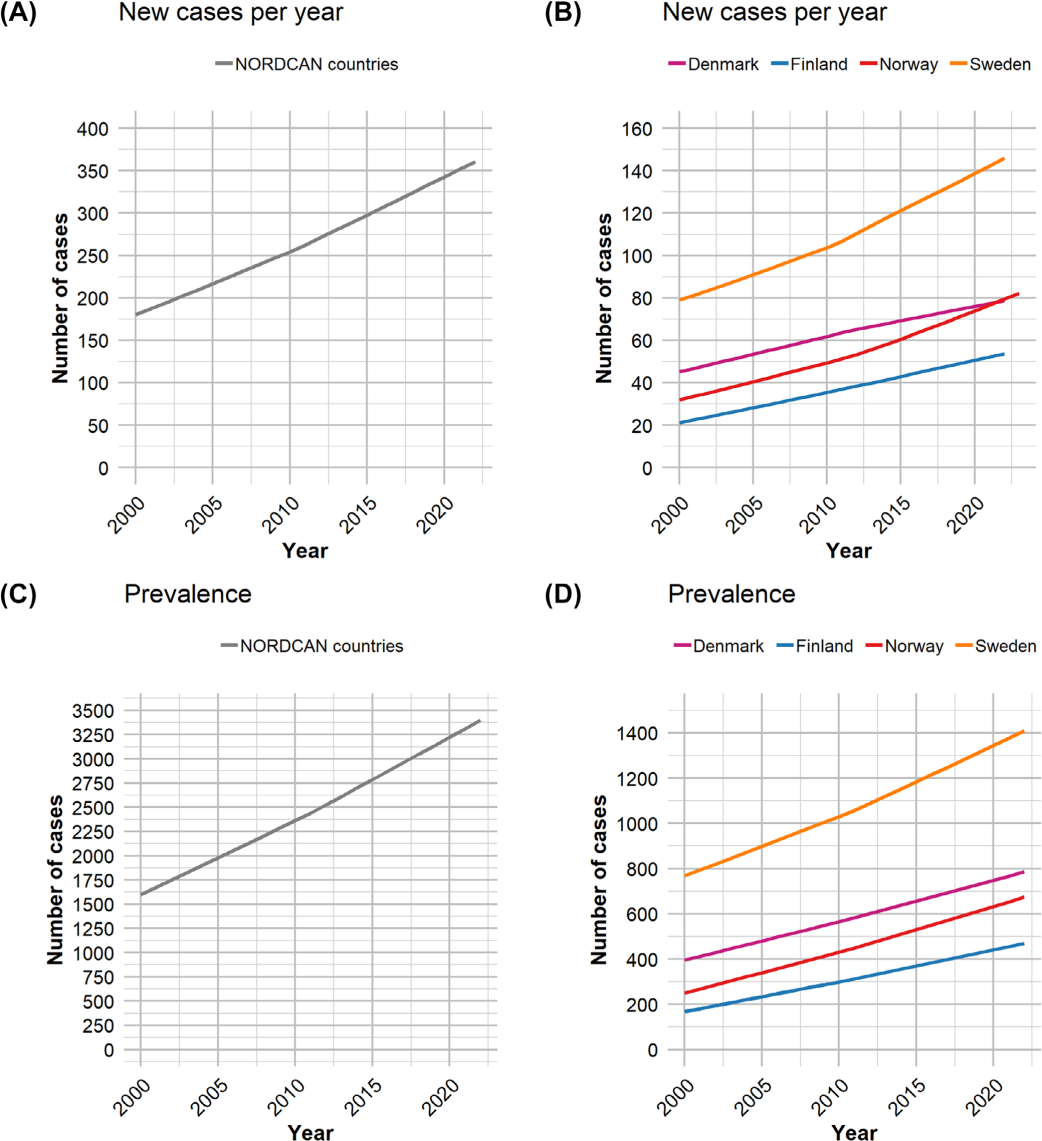
(A) Number of new penile cancer cases in the Nordic countries. (B) Number of new penile cancer cases for each country. (C) Number of patients living with penile cancer in the Nordic countries. (D) Number of patients living with penile cancer for each country. All panels show the yearly trend over the period 2000–2022.

The number of new diagnoses of penile cancer has approximately doubled over the period (Figure 1A), with increasing numbers across all countries (Figure 1B). Currently, more than 300 individuals are diagnosed with penile cancer in the Nordic countries each year. A similar change is also observed for prevalence, with approximately 3500 men now living with a diagnosis of penile cancer in the Nordic region (Figure 1C,D).

**FIGURE 2:**
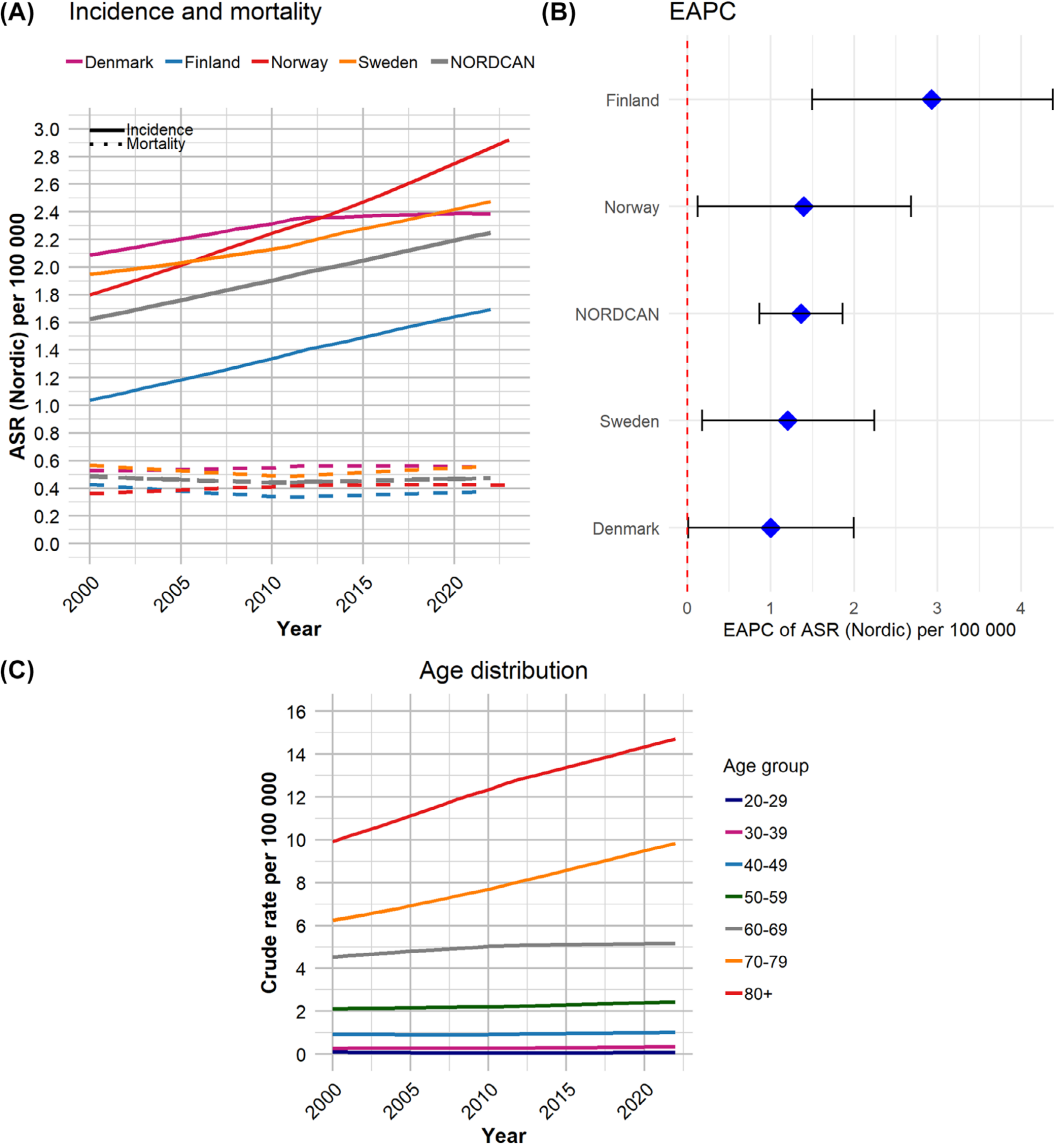
(A) Trend for the incidence and mortality of penile cancer for the Nordic countries combined (NORDCAN) as well as for each individual country. Solid lines represent incidence and dashed lines represent mortality. ASR (Nordic) = age‐standardized rate using the Nordic population as a reference. (B) Estimated annual percentage change (EAPC) of the ASR (Nordic) for the Nordic countries combined (NORDCAN) as well as for each individual country. (C) Crude rate of penile cancer per 100 000 men in the Nordic countries stratified by age groups. All panels show the yearly trend over the period 2000–2022.

The incidence of penile cancer has also increased across all countries (Figure 2A), with a significantly positive EAPC for all countries/regions over the period 2003–2022 (Figure 2B). The ASR of the NORDCAN countries has now moved past 2.0 per 100 000 men. When stratified by age group, data on the number of cases per 100 000 men in the NORDCAN countries show that penile cancer incidence has mainly increased for the 70+ age groups (Figure 2C). Mortality, however, remained unchanged throughout the period (Figure 2A).

**FIGURE 3:**
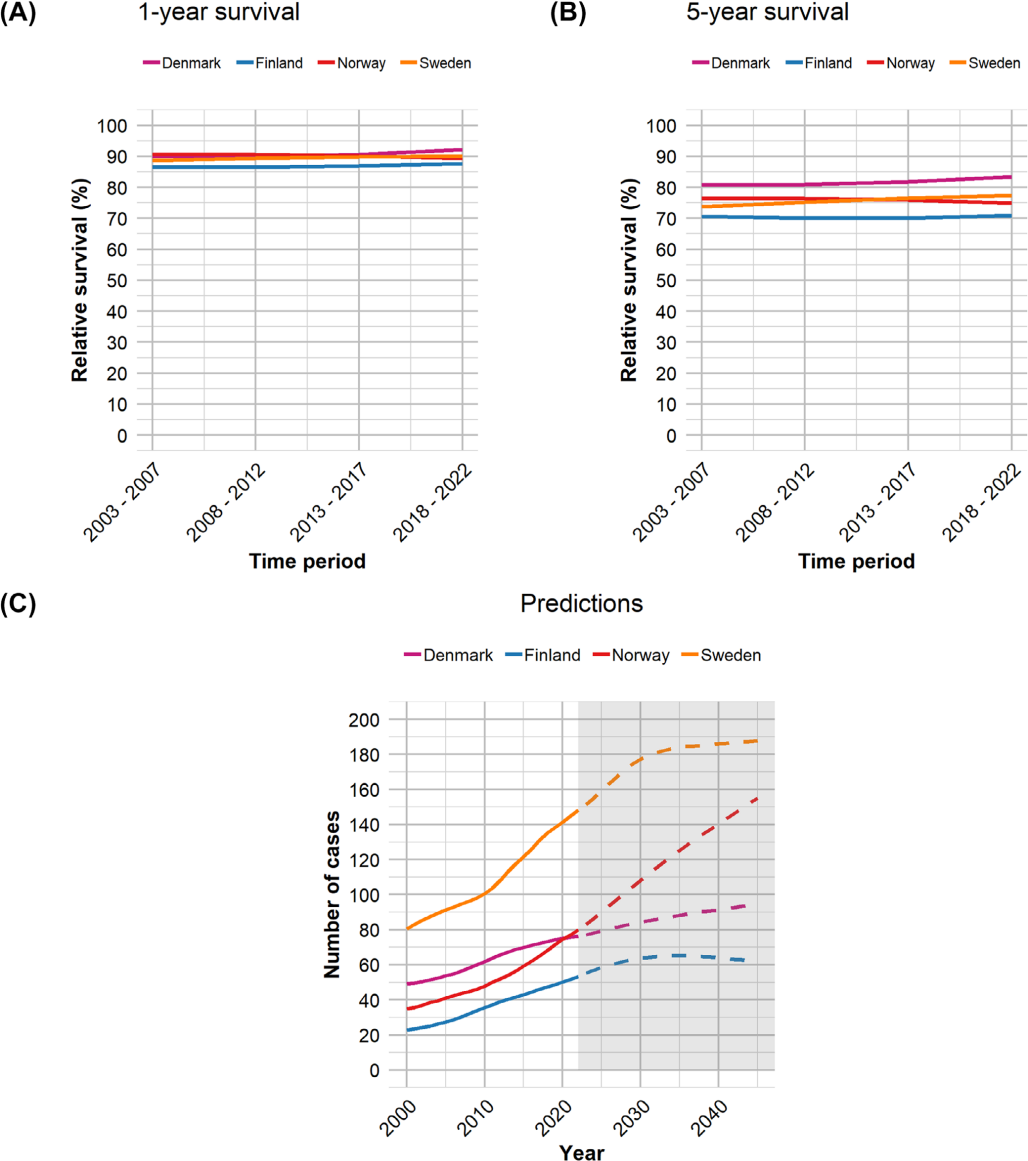
(A) Relative 1‐year survival for patients with penile cancer in each of the Nordic countries over the period 2003–2022. (B) Corresponding 5‐year relative survival. (C) Predicted yearly number of new penile cancer cases by the NORPRED model for each country over the next 20 years.

One‐year (Figure 3A) and 5‐year (Figure 3B) relative survival for the patient group have remained nearly unchanged for all countries over the period 2003–2022.

Based on the NORDPRED model, the yearly number of new penile cancer cases will continue to rise above current levels for all countries over the next 20 years (Figure 3C).

## 4 DISCUSSION

In this epidemiological study, we present data for penile cancer from the NORDCAN database. The findings show that the trend for the yearly number of new cases, as well as for incidence and prevalence of this disease, has increased over the last 20 years in the Nordic countries. Currently, about twice as many patients are diagnosed with penile cancer compared with 20 years ago, and this number is predicted to further increase over the next decades. Mortality and survival have, however, remained nearly unchanged over the study period.

Penile cancer is a devastating and progressive disease where almost all cases eventually present with symptoms. Once diagnosed, reporting to the cancer registries has been mandatory in all Nordic countries during the study period.^5^ The increased number of patients over the period could be explained by several factors. First, the data show that penile cancer has increased mainly among patients older than 70 years of age. With a Nordic population that over time has grown older, people aged 70 years or more now make up just about the largest age group in all these countries. For a disease traditionally occurring late in life, an ageing population could therefore account for at least part of the observed findings. In studies reporting on penile cancer from the Nordic countries, the average age of patients has been around 70 years.^12^, ^13^, ^14^ However, most likely, future studies from the Nordic region will find an even higher mean age for these patients.

Second, in a retrospective cohort study from Norway, it was found that the proportion of cases attributable to infection with human papillomavirus (HPV) had increased over the last 50 years.^12^ Although limited by the retrospective design, similar findings have also been found for head and neck as well as for vulvar cancer.^15^, ^16^ Increasing societal exposure to HPV, likely driven by a higher number of sexual partners in recent decades, could therefore also partly explain the results.

Third, both a Danish and a Swedish study have found an increasing incidence of penile intraepithelial neoplasia (PeIN), which is widely regarded as a precursor lesion of penile cancer.^17^, ^18^ Interestingly, both these studies found a twofold increase in PeIN cases over a similar period as in this study. Development of penile cancer from a larger pool of men with premalignant lesions could therefore also in part explain the findings.

Fourth, it is also possible that centralization of penile cancer care has led to better registration and reporting to the cancer registries. However, to what extent these factors individually have affected the changes in incidence should be studied further in future studies. Of note, falling rates of infant circumcision have previously been proposed as a possible contributor to the rising incidence of penile cancer observed in England.^19^ However, this is less likely to apply in the present study, as most men in the Nordic region have historically been uncircumcised.

In a Norwegian penile cancer cohort study with known causes of death, the Pohar Perme net survival estimator yielded excellent results compared to the traditional cancer‐specific survival estimate by the Kaplan–Meier method.^12^ The nearly unchanged 1‐ and 5‐year relative survival estimates over the period are therefore reliable. In general, patients that are treated before the cancer has metastasized have an excellent prognosis.^20^ However, once the cancer has spread, the prognosis is more uncertain regardless of any current treatment regime.^12^, ^20^ In fact, in a study based on data from the Norwegian cancer registry, no significant changes in 5‐year relative survival were found over the period 1956–2015.^21^

To improve penile cancer care, several measures have been established over the period studied. Centralization of care in all Nordic countries as well as limited time from referral to first examination by a dedicated urologist (in a matter of a few days) has been introduced.^22^ National guidelines, based on the European guidelines,^1^ have also been established. All countries now make use of preoperative radiological staging,^14^ as well as the dynamic sentinel node biopsy (DSNB) technique for surgical staging of clinically node negative (cN0) patients.^13^, ^23^, ^24^

While all these measures are important adjuncts for the optimal treatment and future quality of life of these patients, the findings show that they have not translated into a clear improvement in short‐ or long‐term relative survival for this patient group as a whole. Ayres et al.^19^ examined the impact of the centralization of penile cancer care in England, implemented in 2002, using data from 1990 to 2009. The main findings included a rising incidence of the disease, while mortality and both 1‐ and 5‐year relative survival remained stable in the early years following centralization. Vreeburg et al.^25^ investigated the effects of centralization in the Netherlands over a longer period (1990–2020) and also reported an increasing incidence. However, their study found that centralized care was associated with improved 5‐year relative survival. It is therefore possible that the prognostic impact of centralization in the Nordic region will become clearer with longer follow‐up. Also, as the NORDCAN database does not contain information on the distribution of the TNM stages over time, the effect of a potential stage migration over the period cannot be accounted for in the interpretation of the survival data. Of note, however, a recent Swedish study showed that after centralization of care in 2015, adherence to the guidelines had significantly improved, and more patients with advanced stages of penile cancer now receive additional oncological treatment (i.e. radiotherapy and/or chemotherapy) with a resulting improvement in cancer‐specific survival.^26^

Based on the NORDPRED‐model, there will be more patients diagnosed with penile cancer in the Nordic countries in the coming years. In an effort to produce long‐term preventive effects, school‐based free HPV immunization programmes for boys have been introduced across all the Nordic countries over the period 2018–2023. While this is too recent to have had significant impact yet, it may affect the projected number of new cases over the coming decades. The current situation, however, underscores the need for additional prompt interventions. First, it highlights the need for allocation of more resources into penile cancer care in the region. Second, this calls for public health initiatives to increase public awareness of penile cancer, in an effort to drive earlier diagnosis and treatment. Third, more funding and research to obtain better treatment options for these patients are urgently needed. Studies on the role of immunotherapy for the treatment of advanced penile cancer are underway.^27^ Currently, however, the exact role of immunotherapy is not clear, and early results show that the effect may be better in patients with HPV positive tumours.^3^, ^28^ Another interesting approach is the use of Next‐generation sequencing (NGS) mutational analysis of tumour tissue, which can reveal actionable mutations that can be specifically targeted in the treatment of advanced penile cancer.^29^ Currently, however, NGS analyses are used only infrequently for this patient group in Europe.^30^ More research into this method is therefore needed before it can become part of the clinical routine for these patients. It is also possible that using NGS analyses for detection of actionable mutations combined with measurement of circulating tumour DNA (ctDNA) for disease monitoring may provide a future personalized treatment strategy.^31^ Several approaches are thus underway to further improve survival for these patients. Given the rarity of this disease, including these patients in prospective trials should therefore always be considered.

The NORDCAN database is limited by the fact that no information on histopathological findings or stages is included in the data. Moreover, there is no information regarding important characteristics such as HPV or p16^INK4a^ status of the tumours.^1^ Additionally, the database lacks information regarding the type of treatment offered for individual cases. There is no information on the proportion of patients with different risk factors for the development of penile cancer. As such, the data cannot be used to study why there are differences in incidence between different countries/regions. However, the results from NORDCAN are strengthened by the fact that nearly 100% of the cases have been verified by histopathological examination, as well as the possibility of investigating all the common epidemiological variables in detail.^5^ Moreover, the incidence data in this study has been presented using the Nordic population as a reference, to best reflect the burden of penile cancer in the actual population under study. However, the NORDCAN database also includes incidence data based on the European or the world as a reference population, for easy comparisons against other regions.

## 5 CONCLUSIONS

Penile cancer incidence and prevalence have increased in the Nordic region over the last 20 years. Mortality and survival, however, have remained unchanged. Based on current data, it is predicted that the number of new penile cancer cases will continue to increase in these countries over the next decade. Better treatment options for these patients are therefore urgently needed.

## AUTHOR CONTRIBUTIONS

**Christian Arvei Moen:** Conceptualization; data curation; formal analysis; investigation; methodology; project administration; software; validation; visualization; writing—original draft; writing—review and editing. **Axel Gerdtsson:** Methodology; validation; writing—original draft; writing—review and editing. Mikael Aagaard: Methodology; validation; writing—original draft; writing—review and editing. **Andreas Hopland:** Methodology; validation; writing—original draft; writing—review and editing. **Peter Kirrander:** Methodology; validation; writing—original draft; writing—review and editing. **Jakob Kristian Jakobsen:** Conceptualization; formal analysis; investigation; methodology; validation; visualization; writing—original draft; writing—review and editing.

## CONFLICT OF INTEREST STATEMENT

Jakob Kristian Jakobsen has a research grant from the Novo Nordisk Foundation, received travel support and has a sponsored research agreement with Medac and did consultancy work for Cystotech. None of the other authors report any conflicts of interest.
